# Preferences of young adults on the development of a new digital add-on alcohol intervention for depression treatment: A qualitative study

**DOI:** 10.1016/j.invent.2023.100641

**Published:** 2023-06-30

**Authors:** Maria J.E. Schouten, Marloes E. Derksen, Jack J.M. Dekker, Anna E. Goudriaan, Matthijs Blankers

**Affiliations:** a Arkin Mental Health Care, Department of Research, Amsterdam, the Netherlands; b Vrije Universiteit Amsterdam, Department of Clinical, Neuro- and Developmental Psychology, Amsterdam, the Netherlands; c Amsterdam Public Health, Digital Health & Mental Health, Amsterdam, the Netherlands; d Amsterdam UMC, location University of Amsterdam, Department of Medical Informatics, eHealth Living & Learning Lab, Amsterdam, the Netherlands; e Amsterdam UMC, location University of Amsterdam, Department of Psychiatry, Amsterdam Institute for Addiction Research, Amsterdam, the Netherlands; f Trimbos Institute, Netherlands Institute of Mental Health and Addiction, Utrecht, the Netherlands

**Keywords:** Depression, Problematic alcohol use, Digital intervention, Young adults, Treatment, Focus groups

## Abstract

**Aim:**

To explore the preferences of young adults with regard to the development of a new digital add-on alcohol intervention to complement depression treatment.

**Methods:**

This qualitative study included young adults (18–35 years) with experience of either problematic alcohol use or depression or both (*n* = 29). Two rounds of focus groups were conducted, with two focus groups in each round. All focus groups were recorded, transcribed and analysed deductively and inductively on the basis of qualitative content analysis of the intervention type, features and design.

**Results:**

Young adults preferred a mobile health application with a clear and simple objective and navigation which was also accessible on a computer. With regard to intervention features, participants indicated a preference for in-depth, gain-framed information on alcohol use and a main feature enabling them to record their alcohol use and mood, which would be rewarded. Other preferences included personal goal-setting and monitoring, an activity list, experience stories, peer contact, guidance from experts by experience or volunteers and receiving notifications from the application. In terms of design, participants preferred short, animated videos and animation figure illustrations to complement written text. Moreover, participants rated the design of the intervention as highly important, yet very personal. Generally, participants preferred a light pastel colour scheme. Once again, participants indicated a need for a clear dashboard using pictograms to reduce the amount of text and fast, easy-to-use navigation.

**Conclusion:**

The preferences indicated by young adults with regard to the intervention type, features and design may enhance the development of a new digital add-on alcohol intervention to complement depression treatment.

## Highlights

• Young adults' preferences for a digital alcohol intervention were explored.
• Young adults mainly agreed upon following preferences:
• They preferred a mobile application which was also accessible on a computer.
• Preferred features were recording alcohol, mood, personal goals and peer contact.
• Design preferences included animated videos, illustrations and light pastel colours.

## 1 Introduction

Depression and problematic alcohol use (including alcohol use disorder (AUD) and non-clinical forms of hazardous drinking) are common and often co-occur among young adults. This co-occurrence has been associated with increased risk of suicide attempts, alcohol dependence and poorer global functioning and life satisfaction compared to either condition alone (Pedrelli et al., 2016; Briere et al., 2014). Given these negative outcomes, early intervention is important.

In the United States, treatment use for a major depressive episode (MDE) increased significantly between 2011 and 2019 among young adults (18–25 years), whereas AUD treatment use has increased far less among this age group with co-occurring MDE and AUD (Lu et al., 2022). Moreover, treatment use for both conditions has stayed stable, with <9 % of young adults with co-occurring MDE and AUD being treated for both conditions during 2011–2019 (Lu et al., 2022). These treatment use trends may be attributed to various factors from both a health provider and consumer perspective. For example, health professionals might experience difficulties in identifying co-occurring alcohol problems among young people with depression (Lubman et al., 2007). Consequently, alcohol use problems may remain undetected and untreated. According to Lu et al. (2022), another explanation may lie in a potential mismatch between this young population and current AUD treatment. Young people may not relate to existing AUD programmes, as these often seem to be aimed at and attended by older adults (who are in different life stages) or people with more severe AUD (Kelly et al., 2008; Lu et al., 2022). Moreover, barriers for mental health help-seeking may also play a role, as young adults may not be ready to stop drinking or may lack transportation to attend programmes, fear negative opinions on the part of others or experience stigma and prefer to solve problems alone (Lu et al., 2022; Wu et al., 2007; Ebert et al., 2019). It is therefore crucial to improve treatment for young adults with co-occurring depression and problematic alcohol use. By tailoring the treatment (content) to young adults and providing digital treatment options, treatment may become more accessible for this young population.

Given that young adults experience stigma and prefer to solve problems alone, digital self-help-based interventions seem especially suitable for young adults according to Ebert et al. (2019). Potential benefits of digital self-help interventions include: 24/7 accessibility, large reach, perceived anonymity and a tailored approach. Tailoring can include self-paced programme completion, information preferences (i.e. videos or text) and personalization in when, how or how often to use the programme (Van't Hof et al., 2009; Olff, 2015). Moreover, combined digital interventions targeting both alcohol use and depression also seem promising for adults with these co-occurring conditions, as meta-analytic evidence shows preliminary positive results (Schouten et al., 2022; Riper et al., 2014). A digital self-help-based intervention tailored specifically to young adults with co-occurring depression and problematic alcohol use may therefore be a promising next step. However, there is a relative dearth of qualitative studies on tailoring digital alcohol interventions specifically to young adults with co-occurring problematic alcohol use in depression treatment. To our knowledge, only one study reports on the development of general-population, internet-based self-help for young adults with co-occurring depression and problematic alcohol use (Deady et al., 2014).

Despite their potential, digital interventions tend to have low adherence and engagement, and consequently lower effectiveness (Ramos et al., 2021; Molina-Recio et al., 2020). These challenges might be related partly to the common minimal involvement of end-users in the design process (Eyles et al., 2016). If a user-centred design approach is adopted instead, end-users provide their input throughout the design process, often when determining intervention requirements and conducting usability evaluations. In this way, ease of use, functionality, cost and safety can be established (Blankenhagel, 2019; Molina-Recio et al., 2020). A meta-analysis of randomized controlled trials on serious games for health lifestyle promotion showed the benefit of taking a user-centred approach. The roles of users (e.g. co-design and informants) and design elements (e.g. dynamics or challenges) influenced effectiveness (Desmet et al., 2016). Including end-users in digital intervention design thus appears crucial to meet their specific needs and can enhance intervention adherence, engagement and effectiveness.

Given the relative scarcity of suitable digital treatments specifically for young adults with co-occurring depression and problematic alcohol use, more tailored digital interventions are warranted (Schouten et al., 2022). As part of a larger research project, we developed a new add-on digital alcohol intervention to complement depression for young adults with co-occurring depression and problematic alcohol use (for details, see Schouten et al., 2021). The current qualitative study was conducted using focus group data collected among young adults to inform the development of this digital alcohol intervention. Our research question was: What are the preferences of young adults with regard to the intervention type, features and design of a new digital add-on alcohol intervention to complement depression treatment?

## 2 Material and methods

### 2.1 Randomized controlled trial

This qualitative study was conducted to inform the development of a novel add-on digital alcohol intervention to complement depression treatment for young adults with co-occurring depression and problematic alcohol use. The development of the digital alcohol intervention was part of a larger study in which we aimed to develop and evaluate the (cost-)effectiveness of this digital alcohol intervention for depression treatment (Schouten et al., 2021). The randomized controlled trial protocol and detailed overview of the content and features of the final digital intervention have been published elsewhere (Schouten et al., 2021). The add-on digital alcohol intervention was developed to be followed simultaneously, but not integrated with, regular outpatient depression treatment (treatment as usual, TAU). TAU often consists of any type of evidence-based psychotherapy (e.g. cognitive behavioural therapy, interpersonal psychotherapy or problem-solving therapy) provided by, for example, a therapist, general practitioner or general practitioner mental health worker. TAU could be delivered face-to-face, blended (combination of face-to-face and digital) or exclusively digitally through video calls.

### 2.2 Study design

The current study had a qualitative study design. We conducted focus groups in order to explore young adults' preferences for a new digital alcohol intervention to complement depression treatment. This study was submitted for ethics review and approved by the Scientific Research Committee of Arkin Mental Health Care (Amsterdam, the Netherlands) before commencing the study. All participants provided written informed consent after being informed (both verbally and in writing) about the study's objectives and participation in the focus groups.

### 2.3 Recruitment

We employed both online and offline participant recruitment strategies. Participants were recruited through information flyers which were sent to (youth) support groups and therapists and distributed in waiting rooms of the participating mental health care sites of Arkin and GGZ inGeest in Amsterdam, the Netherlands. Social media recruitment included Facebook audience network advertisements (e.g. Facebook, Instagram, and Messenger). All potential participants were invited to complete an online questionnaire with information on the study and gave consent for questionnaire data collection. Potential participants then completed a background questionnaire including the AUDIT-C (Alcohol Use Disorder Identification Test-Concise) and questions on sociodemographic factors and contact information (Bradley et al., 2007). Participants were eligible to participate if they were between 18 and 35 years old and had any experience of depression (treatment) and/or alcohol use. All eligible potential participants were telephoned by a research team member (MS) to validate their focus group application. Applicants still interested in participating in the study received practical and study information together with the informed consent form by e-mail.

### 2.4 Procedures

We conducted two rounds of focus groups, with two focus groups in each round. The first round was aimed at exploring the preferences for the development of an add-on digital alcohol intervention for depression treatment. Eight participants from the first round of focus groups were also invited to participate in the second round. In the second round we focused on collecting direct feedback on the protype of the digital intervention. The first round (1A and 1B) took place on 15 and 16 August 2018 and the second round (2A and 2B) on 9 and 11 July 2019. The time between the rounds was used to develop a prototype of the digital intervention that could be demonstrated in the second round. All focus groups were moderated by an independent researcher (not involved in the project) and an assistant moderator (MS). We started every focus group by informing the participants about the study and the goal of the focus group. After participants signed the informed consent form, audio-recording devices were turned on. The focus groups were conducted according to a semi-structured topic guide focused mostly on preferences regarding the design and content of the digital intervention (see Table 1). We used a PowerPoint presentation to display the specific topic that was being discussed and to provide participants with examples (e.g. different potential designs). Every focus group lasted 2 h and participants received a €40.00 gift voucher.

**Table 1:** Topic guides.

Focus groups	Round 1	Round 2
Topic guide	Preferences regarding: • Delivery method: app, website or web app • Programme goals: alcohol moderation, abstinence and increasing knowledge of risks and consequences of alcohol use • Potential themes for module content: alcohol refusal skills, dealing with craving, student-related content or other themes • Integration of depression and depression treatment: how and to what extent • Improving motivation and intervention usage • Peer contact and form • Intervention design: “look and feel” concerning different designs • Guidance: by whom, what type, delivery method and how often • Information delivery method: text, video (animation or real person) • Framing: emphasize the risks of alcohol use or the benefits of reducing alcohol use	Feedback on prototype regarding: • Colour scheme: colours and brightness • Dashboard: user-friendly • Alcohol and depression-related assignments: tone, length and content of text • Quiz questions at end of every module • Programme features, e.g.: personal activity rating list, registering of alcohol use, experience stories • Design animation video • Forum board topics • Notifications

### 2.5 Data analysis

The focus group audio was transcribed verbatim. The authors MS and MD then iteratively analysed all data individually by means of qualitative content analysis using MAXQDA 2022 (VERBI Software, 2021). Data analysis began deductively using a preliminary code tree based on the topic guide (i.e. intervention type, intervention features, design and content). Data analysis continued through inductive open coding. Here, newly appeared codes were added to the code tree and applied to all data. Finally, axial and selective coding was applied, in which relationships between codes were disclosed and core- and sub-themes established (Boeije, 2009). Consensus meetings were held in which MS and MD agreed on the majority of the coded data and remaining disagreements were resolved. Pursuing data analysis reflexivity, recurring research meetings among authors were held. To report results of the focus groups, the Standards for Reporting Qualitative Research were used (O'Brien et al., 2014).

## 3 Results

### 3.1 Participants

A total of 29 participants participated in the focus groups. The first two focus groups were conducted with seven and eight participants in each group. The second round of focus groups was attended by eight and six participants, four of whom also attended one focus group from the first round, resulting in a total of 25 unique participants. The mean age was 26.8 years and 76.0 % of the participants were female. Most participants had completed high (44.0 %) or intermediate (40.0 %) education and 36.0 % were currently still studying. All participants had experience of alcohol use and the mean AUDIT-C score was 6.5 (SD 2.5), indicating increased risk of hazardous drinking. Most participants had already reduced their alcohol use (44.0 %) or were contemplating doing so (48.3 %). The majority of participants had experience of alcohol use and depression treatment (84.0 %). Most (48.0 %) had already finished depression treatment and 32.0 % were currently receiving treatment for a depressive disorder (see Table 2).

**Table 2:** Characteristics of the study sample.

	Study sample	
	(*n* = 25^a^)	
	N	%
Sociodemographic characteristics		
Female gender	19	76.0
Age [mean, sd]	[26.8	4.7]
Education		
Low	4	16.0
Intermediate	10	40.0
High	11	44.0
Vocational status		
Student	9	36.0
Paid job	4	16.0
Looking for a job	5	20.0
Other (e.g. sick listed, social welfare benefits)	7	28.0
Background		
Dutch	20	80.0
Non-western migration	4	16.0
Western migration	1	4.0
Country of birth		
Netherlands	23	92.0
Other	2	8.0
Clinical characteristics		
AUDIT-C total score [mean, sd]*	[6.5	2.5]
Alcohol use reduction		
Reduced alcohol use	11	44.0
Contemplating reducing alcohol use	12	48.0
Not contemplating nor reduced alcohol use	2	8.0
Depression treatment		
Currently in treatment	8	32.0
Completed treatment	13	52.0
Not (yet) in treatment (e.g. waitlisted)	4	16.0
Experiences of alcohol use and depression treatment		
Alcohol use and depression treatment	21	84.0
Only alcohol use	4	16.0

a In total 29 (25 unique) participants participated in the focus groups; *AUDIT-C (0−12); a total score of ≥3 (women) or ≥4 (men) indicates at least elevated risk of hazardous drinking (Bradley et al., 2007).

### 3.2 Focus group results

Data analysis resulted in three core themes, namely preferences of young adults regarding 1) intervention type, 2) intervention features and 3) design of a new digital add-on alcohol intervention to complement depression treatment.

#### 3.2.1 Intervention type preferences

Most participants expressed a need for a tailored digital alcohol intervention, as this was currently non-existent in Dutch mental healthcare. Some stated that alcohol use was often overlooked in depression treatment and that paying attention to alcohol use also led to more recognition, which was viewed as positive. Others stated that a digital alcohol intervention could also provide support and a sense of solidarity. One participant felt that the current availability of alcohol intervention websites focused only on older age groups. Another participant expressed a preference for face-to-face contact rather than digital interventions.

“Yes, well, I sometimes think, yes, it might sound silly…. but when you can't call anybody, and you look online and you see these websites and… they address you so formally, it's just for different age groups.”

Focus group 1A, participant 5

“I think the idea that you're not alone, and that you have some sort of support. Especially if you have things, things that you'll monitor, like drinking less or drinking a lot, because nobody ever tells you to monitor that. And something like that helps, it can also be a motivator, like “Oh it's been a long time since I drank [alcohol]…”

Focus group 1B, participant 13

Participants expressed a preference for a clear and simple intervention objective. They mentioned wanting to understand why they drank and on what occasions. They also said they wanted autonomy to personalize the intervention objective, either to stop drinking or to reduce their alcohol intake.

“That you also gain insight into your drinking behaviour, and when and why.”

Focus group 1A, participant 4

Participants preferred the intervention to be delivered as a mobile health application, mainly because they saw it as the most accessible option. They explained that mobile health applications are easy to use, they always have their smartphone with them and the intervention would always be available. In addition, mobile health applications allow notifications. On the downside, however, a participant mentioned that a mobile health application required downloading, which could be a barrier for users with a lot of applications and little free memory.

“It's nice if the user threshold is very low, which it generally is on an app that you can easily use on your phone.”

Focus group 1A, participant 4

With regard to a website, participants mentioned that it would be nice to access the intervention on a computer, as a larger screen would likely facilitate intervention use. On the other hand, adherence to the intervention would likely be lower, as they perceived it to be harder to maintain or to remember to interact with the website. A combination of an intervention accessible by mobile phone and computer, i.e. a web application, was considered ideal, as participants could have the advantages of both.

“Nowadays there are a lot of computers that can, erm, on which you can also access mobile applications, right? Because if that's possible, it would be ideal! Because if I wanted to [follow the programme] I would maybe want it on a larger screen because it's easier to read.”

Focus group 1B, participant 13

#### 3.2.2 Intervention features

##### 3.2.2.1 Modules

Participants said they wanted a clear, manageable intervention. For example, with regard to an overview of intervention modules, they wanted modules to be named on the basis of content rather than being numbered. Views differed on the addition of a quiz at the end of a module to check attainment of learning goals. While some participants liked quizzes, others found them pedantic. As an alternative, a self-reflection assignment was suggested.

“I think you'll get a reprimand if you haven't read it properly.”

Focus group 2B, participant 7

Participants were inconclusive about the set five days of practice before continuing to the next module. On the one hand, they said they would feel ‘on hold’, which annoyed them. On the other hand, they explained that it would offer stability, as emotions come and go during depression and the ‘waiting time’ forces them to go through the intervention.

“I understand it's very frustrating, I want to move on. I'm on hold for five days.”

Focus group 2B, participant 4

“There's also something good about being forced to wait. (…) When you have to wait, you're very aware of what you're doing.”

Focus group 2B, participant 3

##### 3.2.2.2 Information provision

A need for information on the risks of alcohol use was expressed in the focus groups. Participants expressed a preference for in-depth information that was gain-framed (e.g. physically or financially). Some participants also wanted information to be included on other mental health disorders or drug use in the intervention, while others feared that by including such information the intervention might lose focus and become unclear.

“Truly focused on the positive aspects [i.e. effects on reducing/stopping alcohol consumption], indeed that's really important”

Focus group 1B, participant 14

##### 3.2.2.3 Monitoring alcohol use and mood

One of the main intervention features according to participants should be the recording of their alcohol use (i.e. how many drinks, where and with whom). Participants explained that recording their alcohol use enabled them to gain insight into their use and monitor their progress, for example by means of graphs. Participants wanted to be rewarded for recording their use (e.g. with badges), regardless of whether they met their (daily) goal. Participants said this would encourage them to record honestly and not to feel too bad if they failed to meet the goal once. That is also why participants explicitly did not want a run streak feature (i.e. tracking the number of completed alcohol consumption recordings consecutively, without fail).

“For me, the core goal would be to really get that alcohol use under control. I would use the app mainly for that. Really focused on that.”

Focus group 1A, participant 4

“I would really like it if it said ‘well done’ every time you filled it in at all.”

Focus group 1B, participant 12

Alongside monitoring their alcohol use, participants expressed the desire to monitor their mood simultaneously. Participants said they felt that their mood and alcohol use were intertwined and monitoring both would give them insight into their treatment. To ease their mood monitoring, it was suggested that smiley faces (emojis) could be a simple means of indicating their mood.

##### 3.2.2.4 Goal-setting and activity list

Setting personal goals and monitoring goal attainment was another suggestion by participants. Participants wanted to link this to a readily available ‘activity list’. It would serve as a ‘go to’ feature at difficult times or as a means of distraction to keep on track with personal goals. Participants suggested including contact details in the list in case help were needed, as well as pre-installed suggestions for activities and the ability for the user to add activities. Such a list would help with finding alternative activities when participants were having a difficult time.

“Suppose you're not comfortable in your own skin or something. And then you go on that app and look at your profile. ‘What shall I do now? ‘Oh yes, I'll watch a movie or draw.’ Then you actually have a kind of tool.”

Focus group 2A, participant 6

##### 3.2.2.5 Experience stories

Most participants said they would prefer experience stories to be incorporated into the intervention modules, in both short formats (e.g. quotes) and longer formats (e.g. long authentic experience stories). They said such experience stories were more interesting to read, as they add a personal touch to the assignments and otherwise it may feel more like ‘theory’. Experience stories also increase recognition, promote sympathy with others and make people feel less alone and provide them with perspective for the future.

“If you make it [the experience story] more personal, it becomes more interesting. Because now it's not really… it's not really different from what I read on the internet, but if you have such an app [digital alcohol intervention] and all these experience stories of people in it, you feel stronger, I think, because you feel you're doing it together, and you can recognize yourself in it.”

Focus group 2A, participant 4

##### 3.2.2.6 Peer contact

As well as experience stories, participants called for an online community with peers. This was seen as a low-threshold, non-judgemental and motivational way to share feelings or experiences with others, compared to face-to-face groups or non-peers. One participant said a forum, despite having some advantages, also felt somewhat old-fashioned and doubted whether people would still use it. However, most participants opted for a forum rather than a chat as the delivery mode. A chat was seen by many users as too messy and as having greater potential for negativity.

“An app with such a community, I think, would be accessible. (…) You do see people there with questions and people respond to them.”

Focus group 1A, participant 7

“I do think you can ask a sort of help-related question on a forum… and people who want to answer respond, and those who don't want to respond don't. I think you should at least offer it [a forum], and give people the opportunity to use it if they feel like it. Because I believe it can help a lot of people.”

Focus group 1B, participant 11

##### 3.2.2.7 Guidance

With regard to guidance in using the intervention, participants indicated that they would prefer to have support available from experts by experience or a volunteer rather than their depression therapist or a chatbot. The guidance could consist of a positive note, motivation or answers to questions, preferably delivered by a chat functionality.

“I think maybe just an expert by experience. Yes, I like those people very much anyway. Never pedantic or anything. So that's very nice. Certainly for someone who's just taking that step, it's nice to have experts by experience, who are not all psychiatrists.”

Focus group 1B, participant 8

Therapists having access to participants' intervention data (e.g. alcohol consumption records) had both advantages and disadvantages. On the one hand, few participants believed it would aid the integration of the intervention use with the depression treatment. Some participants expected that their therapist having insight into – for example – their mood recording would help them discuss important issues during treatment. However, the majority of participants wanted autonomy in terms of the data they wanted to share with their therapist. At the same time, participants were hesitant about therapists having access to their intervention data, as they feared their intervention input might be socially desirable rather than truthful.

“I could say otherwise, but in reality that's just really the truth. Then I'm not truthfully going to write down how I really feel inside if someone is going to read it.”

Focus group 1A, participant 5

##### 3.2.2.8 Notifications

Participants wanted to receive notifications from the application. They proposed notifications with positive and motivational content, tips and information, and reminders to record their alcohol use or mood. Participants preferred to personalize the content, frequency and timing of the notifications.

#### 3.2.3 Design

##### 3.2.3.1 Animated videos

Participants had a positive view of incorporating short, 1–3-minute, animated videos into the intervention programme. They preferred animated videos to written text, as they come across as stylistic, young and more appealing, but also make psycho-education more accessible and easier to follow. By providing subtitles and the opportunity to read the (extended) content of the video, it is also possible to work through the content of the videos without audio or to re-read information instead of watching the video again.

*“*It quickly becomes very boring to look at, so an animation is more, yes, I think, nicer to look at, a little bit happier.”

Focus group 1A, participant 3

##### 3.2.3.2 Illustrations

Participants preferred alcohol-related illustrations and images not to be patronizing and stigmatizing. They also disliked stock photos and images, as they often seem unrelatable and stereotypical. However, they also preferred not to see realistic and appealing illustrations of alcohol, as these would be too triggering.

Most participants preferred animation figures to complement written psycho-education. A recurring animation figure can make long written texts more appealing but also help with remembering certain psycho-educational topics, as one might remember the illustration and therefore the topic. Participants expressed different views concerning the design of animation figures, but figures that were identifiable, inclusive, stylistic and adult-looking were preferred by most participants. However, some participants also preferred a funny and airy animation figure, such as an animal figure, to maintain a light-hearted feel.

“Yes, it's a little bit childish, but it's also sort of… friendly looking. And I believe what you need is not an app that tells you professionally what's good for you and what happens to your liver if…. but just a cheerful whale that says stop drinking! [laughter]”

Focus group 1B, participant 12

##### 3.2.3.3 Colour schemes

Participants said the design of the intervention was important, as a nicely designed intervention would give the user more confidence about the quality of the programme and may increase programme usage. However, participants said that preferences as to what constitutes a good design (and related colour schemes) are very personal and it might not be possible to please everyone. Generally, participants expressed different views regarding a colour scheme with bright pink and yellow colours against a white background. Some thought it was nice and clear, but the majority thought the bright colours were too feminine, too bright and cheerful, unsuitable for an alcohol programme or drew too much attention and were therefore not discreet enough.

“Well, I've been thinking, when I slip into a depressed mood, I don't feel like using a bright pink app [laughter]. Because sometimes even the brightness of the light on screen of my phone can be too much, so if I slip away, I already have to adjust that. So if [the programme design] includes very bright cheerful colours, I won't use it.”

Focus group 1A, participant 5

A few participants thought colour schemes using bright colours against a darker background were clear, but the majority said they were too dark and gloomy. Most participants also disliked the use of a blue colour scheme, as it would feel too clinical and chilly. Most preferred the use of light pastel colours, as these looked modern, soft and not too bright, yet still colourful.

##### 3.2.3.4 Navigation and programme dashboard

The programme dashboard serves as a homepage, as it provides an overview of the programme components and enables users to navigate further into the programme. Participants disliked cluttered dashboards with too much text making it unclear where the user should navigate to. The dashboard should be clear and straightforward, using pictograms to reduce text and only showing content accessible to them. Adding a symbol of a lock to the modules that are not yet available will not only lead to clarity but also trigger curiosity about the locked modules. Participants also mentioned that discreetness was important and they preferred to see positive things when opening the programme dashboard, such as their personal progress towards achieving their drinking goals or earned ‘gamification badges’.

“Your dashboards should include something that shows progress or something positive, and then you can go on with everything […] That ‘latest achieved badge’ thing on the right corner below is also nice. When you open the programme and immediately see your last achievement, it makes you think: “Oh yeah, I forgot about that achievement!”

Focus group 1B, participant 13

Participants said programme navigation should be user-friendly and focus on ease and clarity. This included navigation through swiping, dropdown menus to reduce scrolling through content and recording daily moods with emojis instead of typing.

#### 3.2.4 Final digital alcohol intervention

**Fig. 1:**
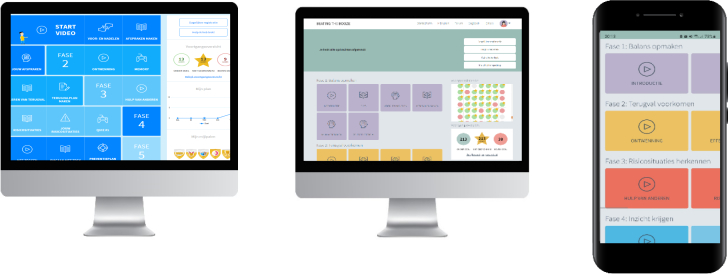
Final digital alcohol intervention. Note: From left to right: base intervention (web-based Jellinek Self-help) and final web-app intervention on computer screen and smartphone.

The digital alcohol intervention was developed through an iterative development process. We developed a prototype web-app intervention based on input from the first round of focus groups and based on the existing web-based Jellinek Alcohol Self-help (Blankers et al., 2011) that had previously been found effective. The prototype was evaluated in the second round of focus groups and, together with input from several addiction and ehealth professionals, final adjustments were made to the intervention, resulting in the final digital intervention. Based on input from the focus groups, various preferences regarding the design (e.g. colour scheme, animation figures) and content (e.g. experience stories) and intervention features (e.g. forum, mood recording, web application) were incorporated into our final digital intervention (see Fig. 1). A detailed overview of the content and features of the final digital intervention has been published elsewhere (Schouten et al., 2021).

## 4 Discussion

### 4.1 Key findings

This study aimed to explore the preferences of young adults with regard to the development of a new digital add-on alcohol intervention to complement depression treatment. Our general findings included the observation that young adults preferred a mobile health application with a clear and simple objective and navigation that was also accessible on a computer. Moreover, participants indicated a need for a clear dashboard with pictograms to reduce the amount of text and a need for fast, easy-to-use navigation. In terms of design, participants preferred short, animated videos and animation figure illustrations to complement written text. Specific findings were that most participants had the same preferences for intervention features including in-depth, gain-framed information on alcohol use and a main feature enabling them to record their alcohol use and mood. Other preferences included personal goal-setting and monitoring, an activity list, experience stories, peer contact, notifications and support from experts by experience or volunteers. Participants were inconclusive about their preferences for or against fixed practice periods before continuing to the next module, information on other drugs or mental disorders and sharing intervention data with their therapist. Participants had different views on the design of the animation figures and the colour scheme of the intervention, which they rated as highly important. Participants mostly preferred a light pastel colour scheme.

### 4.2 Interpretation of findings

#### 4.2.1 Generic findings

Participants in our study said they needed an intervention delivered through both their mobile phone and a web browser. While research into effectiveness and preferences regarding delivery platforms appears in its infancy (Renfrew et al., 2020; Whitton et al., 2015), the literature seems to confirm our finding concerning delivery through mobile platforms in addition to web-based interventions for alcohol and/or depression interventions targeting young adults (Deady et al., 2014; Wright et al., 2016; Whitton et al., 2015). The inclusion of a feature concerning experience stories was highly valued by the participants. This was recognized in earlier studies, indicating that it aids tailoring and is engaging to the young adult population, adding to the relevance of the intervention (Mcdrury and Alterio, 2002; Deady et al., 2014; Kay-Lambkin et al., 2011a; Kay-Lambkin et al., 2011b; Kay-Lambkin et al., 2011). The literature endorses the participants' preference in the current study to be in contact with peers (i.e. adolescents with depression and engaged in substance use) (Lubman et al., 2017; Mason et al., 2019). Our participants appreciated the possibility of online peer support through discussion forums, but also acknowledged that they might not be actively used by all users. Indeed, this often seems to be the case with discussion forums, as Ekström and Johansson (2020) also found that only half of the users of a digital alcohol reduction self-help programme believed discussion forums were useful. Forums seem to attract two types of users: those who are actively involved in posting and interacting with other users and those who are inactive but read the available content (Ekström and Johansson, 2020). Receiving notifications, a preference also expressed by our participants, is known from other studies to increase intervention engagement (Oakley-Girvan et al., 2022). In line with the literature, our findings indicate that the design of an intervention is very important for young people. Young adults seem to have more confidence in the quality of a programme when the design is appealing to them. Moreover, an engaging design (e.g. including animated videos and illustrations) seems to make written texts more appealing and helps to emphasize the core message. Similar findings were also found in other (qualitative) studies on digital alcohol interventions designed specifically for adolescents and young adults (Tinner et al., 2020; Crane et al., 2017).

#### 4.2.2 Specific findings

Our participants indicated a need for a simple and clear intervention, featuring alcohol and mood recording, goal-setting and activity lists, which are features that have also been implemented to positive effect in other combined depression and alcohol interventions for (young) adults (Deady et al., 2014; Deady et al., 2016; Baumgartner et al., 2021). Moreover, participants in the current study called for gain-framed information on alcohol use (i.e. benefits of stopping or lowering alcohol intake) (Rothman and Salovey, 1997), which has been hypothesized to be more persuasive for preventive behaviours such as reducing alcohol consumption (Churchill et al., 2016; Salovey et al., 2002). Evidence on the effectiveness of using gain-framed messages to reduce alcohol intake is inconsistent, however (De Graaf et al., 2015; Pavey et al., 2018; Churchill et al., 2016; Quick and Bates, 2010). The use of animation and images, the use of colours and the addition of quotes were also among the improvements suggested by young adults for a digital intervention for young adults with depression and alcohol use problems (Deady et al., 2014).

### 4.3 Strengths and limitations

The strengths of this study were that multiple focus groups were conducted in different design stages and were well attended. The second round of focus groups included participants from the first round of focus groups as well as new participants to reflect on the implementation of the findings of the first round and generate new insights. However, this study was also subject to limitations. Whilst we expected to approach data saturation, this may have been affected by the predetermined number of focus groups and the re-invitation of former participants. We cannot rule out that additional focus groups or a different composition of focus groups might have elicited other viewpoints. Another limitation concerns data analysis and interpretation likely being affected by subjectivity – although this is integral to qualitative research methods. In order to reduce subjectivity, we added a second, independent coder and held regular research meetings to discuss intermediate results. Finally, users did not actively use the intervention, but were given a walk-through led by the focus group moderator. The preferences identified in this study should therefore not be mistaken for usability findings.

### 4.4 Future directions

Besides taking a user-centred design in developing digital interventions, future studies could consider more extensive usability testing in the development of digital interventions for young adults with co-occurring depression and problematic alcohol use, for instance by giving the end-user a few weeks to use the digital intervention programme as intended or using other usability evaluation techniques (Yáñez Gómez et al., 2014). Such a user experience comes closer to the real user experience and might provide more elaborate feedback on intervention improvements and insights into potential usability. Researchers and developers can use our findings to inform their intervention development. Some design-related preferences (e.g. colour scheme and illustrations) may be more applicable to younger people, whereas depressed populations might favour a design that is not too bright and a feature for recording their daily mood. General preferences such as user-friendliness are relevant to all users. It remains of utmost importance that developers include end-users in any digital intervention development process, particularly since the perceived fit of a digital intervention, i.e. whether an intervention is tailored (e.g. relevant and appropriate) to end-users, seems to be an important factor that can contribute to user engagement, whereas depressive symptoms and low mood might hinder user engagement (Borghouts et al., 2021). This once again stresses the importance of consulting end-users throughout the intervention development process, especially for depressive populations. End-users can identify potential usage barriers in a timely manner, but also bring forward suitable solutions to overcome them, which may improve intervention engagement.

## 5 Conclusions

Young adults preferred a digital alcohol intervention in the form of a mobile application that was also accessible on a computer. Preferred features included (but were not limited to): recording alcohol and mood, personal goals, peer contact, experience stories, support and receiving notifications. An appealing design was deemed important yet very personal, but the use of light pastel colours, illustrations and animated videos was generally preferred. The preferences indicated by young adults regarding the intervention type, features and design can enhance the development of a new, tailored, digital add-on alcohol intervention to complement depression treatment.

## Funding

This study was funded by 10.13039/501100001826ZonMw (the Netherlands Organization for Health Research and Development, grant number 636310009). The funder had no role in the design of the study and will not have any role during its execution, analyses, interpretation of the data or the decision to submit results.

## Declaration of competing interest

The authors declare that they have no known competing financial interests or personal relationships that could have appeared to influence the work reported in this paper.
